# Two mutations in the HR2 region of Newcastle disease virus fusion protein with a cleavage motif “RRQRRL” are critical for fusogenic activity

**DOI:** 10.1186/s12985-017-0851-0

**Published:** 2017-09-25

**Authors:** Yanhong Wang, Youkun Bi, Wanqi Yu, Ning Wei, Wenbin Wang, Qiaolin Wei, Xinglong Wang, Shuxia Zhang, Zengqi Yang, Sa Xiao

**Affiliations:** College of Veterinary Medicine, Northwest A&F University, Yangling, Shaanxi 712100 China

**Keywords:** Newcastle disease virus, F protein cleavage site, Syncytium formation

## Abstract

**Background:**

Newcastle disease virus (NDV) causes severe diseases in avian species. Its fusion protein cleavage site (Fcs) is a major contributor to virulence and membrane fusion. Previous studies showed that a change from phenylalanine (F) to lysine (L) at position 117 of the virulent strain fusion protein, which has the polybasic amino acid Fcs motif “^112^RRQKR↓F^117^”, blocked syncytium formation. However, we observed that F proteins of the virulent strain F48E9 and avirulent strain LaSota substituted with an identical cleavage motif, “^112^RRQRR↓L^117^”, induced extensive and slight syncytium formation, respectively. Accordingly, we hypothesized that the difference in syncytium formation is caused by other regions of the fusion protein.

**Results:**

The exchanged regions between the fusion proteins of two strains, F48E9 and LaSota, showed that the region from amino acid 118–499 plays an important role in modulation of fusogenic activity in transfected cells. Further dissection of this region indicated that replacement of two amino acids (N479D, R486S) in heptad repeat 2 (HR2) of the avirulent fusion protein by the virulent counterpart resulted in fusion promotion. Moreover, the role of these two amino acids in fusion is dependent on the unique Fcs sequence “RRQRR↓L”.

**Conclusions:**

Our results demonstrated that two amino acids (D479, S486) of the virulent strain F protein with this unique Fcs were critical for promoting fusogenic activity, and residue F or L at position 117 did not affect syncytium formation. These findings provide novel insights into fusogenic triggering by the fusion protein and may be useful for designing antiviral peptides.

Newcastle disease is a highly contagious disease caused by Newcastle disease virus (NDV) that results in severe economic losses in the poultry industry. The NDV belongs to the genus *Avulavirus*, family *Paramyxoviridae* and order *Mononegavirales*. It possesses an enveloped, non-segmented, single-stranded, negative-sense RNA genome, which encode six major proteins—the nucleoprotein (NP), phosphoprotein (P), matrix protein (M), fusion protein (F), hemagglutinin-neuraminidase protein (HN) and large RNA-directed RNA polymerase protein (L) [1, 2]. NDV infection of susceptible host cells requires two viral glycoproteins, HN and F. The HN protein mediates attachment to the sialic acid receptor, fusion promotion and release of the virus [3]. The F protein mediates fusion of the virus-cell and cell-cell membranes [4]. Importantly, the F protein cleavage site (Fcs) is believed to be a major determinant of virulence and fusion activity in NDV [5–7]. The F protein is synthesized as an inactive precursor (F_0_), which is cleaved into two disulfide-linked polypeptides, F_1_ and F_2_, by host proteases at the Fcs. This cleavage results in exposure of the fusion peptide at the N terminus of the F_1_ subunit, which triggers fusogenic activity.

NDV strains are generally classified into three pathotypes based on their pathogenicity in chickens: lentogenic (low virulent or avirulent), mesogenic (moderately virulent) and velogenic (highly virulent) [8, 9]. The Fcs of velogenic and mesogenic strains exhibit a polybasic amino acids motif, “^112^R/K-R-Q-K/R-R↓F^117^”, at the C terminus of the F_2_ protein and a phenylalanine (F) at position 117, which is located at the N terminus of the F_1_ protein that is cleaved by ubiquitous intracellular proteases. In contrast, the lentogenic Fcs possesses the monobasic amino acid motif “^112^G/E-K/R-Q-G/E-R↓L^117^” at the C terminus of the F_2_ protein and a leucine (L) at position 117 that is cleaved by extracellular trypsin-like proteases restricted to the respiratory and intestinal tract [10]. Alteration of the Fcs affects the fusion activity and virulence of NDV [6, 11–14]. However, some strains and modified viruses containing an identical Fcs motif present varied fusogenic activity [15–17]. In our study, we observed that mutation of the F proteins of the virulent strain F48E9 and the avirulent strain LaSota to an identical motif, “RRQRR↓L”, altered membrane fusion in transfected cells, suggesting that other regions of the F protein are involved in inducing fusion in addition to the Fcs.

The unique cleavage motif “RRQRR↓L” was found in a natural isolate (99–0868-2) in Australia. Although this isolate had an intracerebral pathogenicity index (ICPI) of 1.38, indicative of a virulent virus, inoculation of the virus into 7-week-old birds demonstrated an avirulent phenotype, suggesting that the motif “RRQRR↓L” is a deduced avirulent Fcs [18]. In our study, we used the F proteins of the virulent strain F48E9 and avirulent strain LaSota as backbones. The F48E9 strain is a standard highly virulent virus in China and possesses a virulent Fcs, “RRQRR↓F”, which induces substantial syncytium formation in the transfected cells. The LaSota strain is a vaccine virus that has an avirulent Fcs, “GRQGR↓L”, which induces slight syncytium formation (Fig. 1a–c). We modified the Fcs of these two strains to the motif “RRQRRL”, yielding F48E9-F* (F48-F*) and LaSota-F* (La-F*) (* represents RRQRR↓L) (Fig. 1a). The expression vector pCAGGS was used for all F and HN constructs [19]. BHK-21 cells were transfected with the F48-F* construct and produced significant syncytium formation when co-transfected with F48E9 HN (F48-HN), whereas La-F* caused slight syncytium formation when co-transfected with LaSota HN (La-HN) (Fig. 1b and c). The BHK-21 cells that were transfected with F48-F, F48-F*, La-F or La-F* alone did not produce syncytia (data not shown). The F and HN proteins of the strains F48E9 (genotype IX) and LaSota (genotype II) are genetically different, with amino acid identities of 92 and 91%, respectively. To exclude the effect of different HN proteins on fusogenic activity, we co-transfected the F48-F* and La-F* constructs with homologous (F48-HN) and heterologous HN (La-HN) proteins, respectively. The results showed that the F48-F* produced extensive syncytia when co-transfected with F48-HN, but larger and more numerous syncytia were observed following co-transfection with La-HN. These findings may be explained by the increased interactions of La-HN with F48-F* compared to those of F48-HN. The amino acid sequences of La-HN and F48-HN, which show 91% identity, are different. In addition, La-HN (577 aa) has an additional six amino acids in the N-terminus, which is longer than that of F48-HN (571 aa). These differences in the two HN proteins may affect their interaction with F48-F*. However, the La-F*-transfected cells produced very weak syncytia when co-transfected with either La-HN or F48-HN (Fig. 1b and c). The syncytia were quantified by counting the number of nuclei in 40 fusion areas to determine the average syncytia size as previously described [20]. The results indicated that the distinct fusogenic activity was due to the F* proteins of two strains but not the HN proteins.

**Fig. 1:**
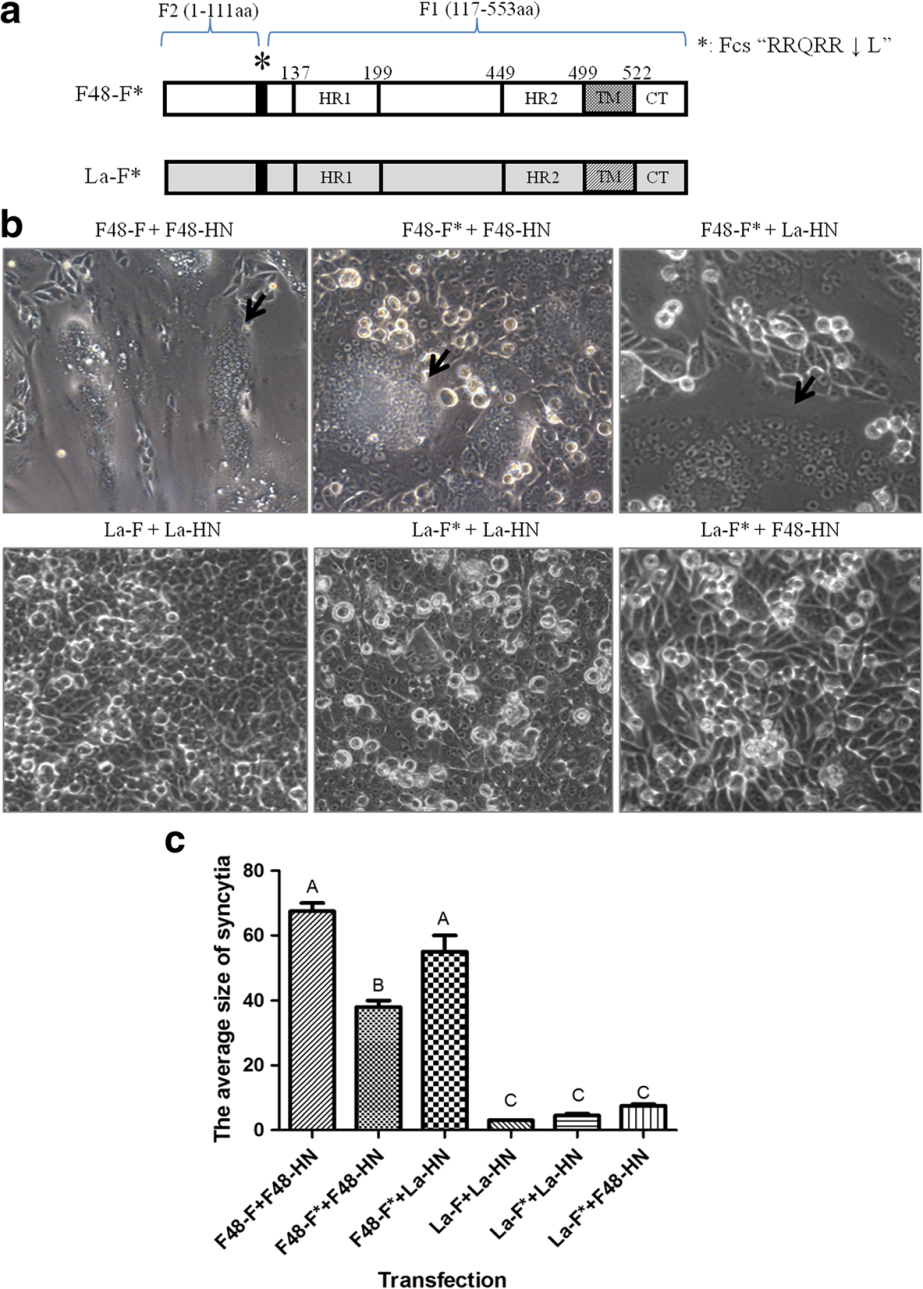
Difference in syncytium formation induced by F48-F* and La-F* in the transfected BHK-21 cells. **a** Schematic diagram of the F proteins of the virulent strain F48E9 and avirulent strain LaSota with the cleavage motif “RRQRRL” (*). **b** Induction of syncytium formation by co-transfection with the F* and HN constructs in BHK-21 cells. At 36 h post-transfection, monolayers were examined for the presence of syncytia, which were photographed under the microscope. Syncytia are indicated by black arrows. **c** Quantitative analysis of the syncytia in (**b**)

To identify which region of the F* proteins is involved in membrane fusion, we substituted the F_2_ subunit (1–111 aa) of the ectodomain (1–499 aa), transmembrane domain (TM, 500–522 aa) and cytoplasmic tail (CT, 523–553 aa) of F48-F* with the corresponding regions of La-F* in the F48-F* backbone (Fig. 2a). Among them, the F48/La1–111, F48/La500–553, F48/La523–553 and F48/La500–522 cells showed extensive syncytia that were similar to those of F48-F* when co-transfected with F48-HN in BHK-21 cells. However, comparatively fewer syncytia were observed with F48/La1–499, F48/La1–522, F48/La118–499, F48/La118–522 and F48/La118–553 (*P* < 0.01) (Fig. 2a and b). The results indicated that the La-F* 118–499 region disrupted the membrane fusion caused by F48-F*. Next, we further replaced the 137–199 (heptad repeat 1 [HR1]), 200–448 and 449–499 (heptad repeat 2 [HR2]) regions of La-F* with the corresponding regions of F48-F*. F48/La137–448 resulted in the most syncytia among these mutants (Fig. 2c and d). Then, the region 449–499 of F48-F* was subdivided into two regions of 449–469 and 470–499. The corresponding mutants F48/La137–448 and F48/La137–469 showed the most syncytia compared to the others (P < 0.01) (Fig. 2c and d), suggesting that region 470–499 of F48-F* played an important role in fusion promotion. There are differences in three amino acids in region 470–499 between F48-F and La-F: aspartic acid (D), serine (S) and glycine (G) in F48-F and asparagine (N), arginine (R) and serine (S) in La-F at the positions 479, 486 and 497, respectively. To identify the role of these three amino acids in fusogenic activity, we substituted these residues of La-F* individually or dually with those of F48-F* on the La-F* backbone. Obvious syncytium formation was observed with La-F*(N479D) or La-F*(R486S) during co-transfection with La-HN in BHK-21 cells but not with La-F*(S497G). The double mutant La-F*(N479D, R486S) produced significantly more and larger syncytia than the single mutants La-F*(N479D) and La-F*(R486S) (*P* < 0.01) (Fig. 3a and b). These results demonstrated that the La-F*-specific residues N479 and R486 played a critical role in fusion disruption.

**Fig. 2:**
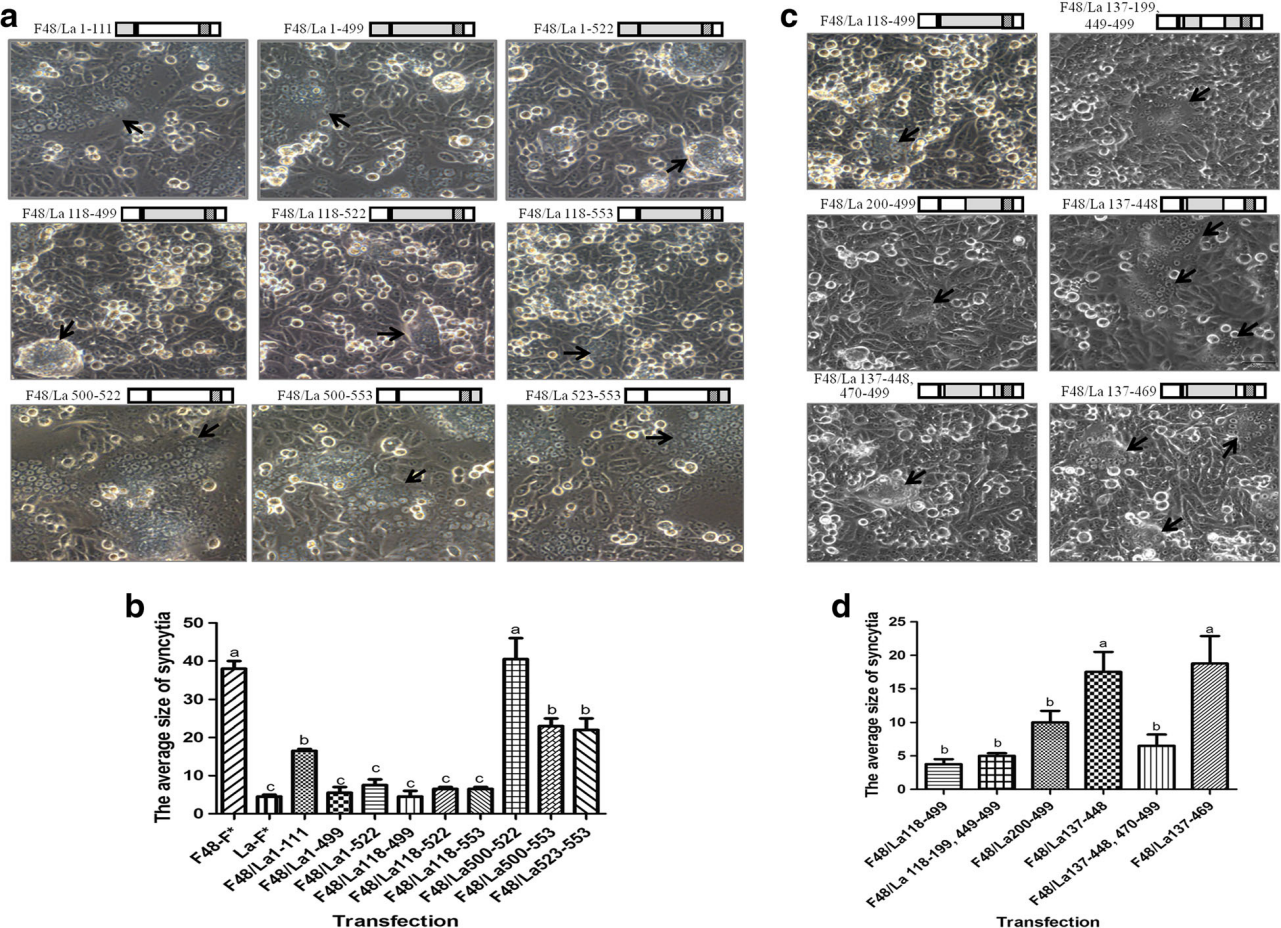
Effect of different regions of the F* protein on membrane fusion. **a** Induction of syncytium formation by different regions between F48-F* and La-F* in BHK-21 cells when co-transfected with F48-HN. Schematic diagram of each construct with substituted regions in F48-F* is shown at the top of each picture. **b** Quantitative analysis of syncytia in (**a**). **c** Identification of region 449–499 of HR2 in triggering syncytium formation. **d** Quantitative analysis of the syncytia in (**c**). Statistical significance is designated with lowercase letters (*P* < 0.05) or capital letters (*P* < 0.01)

**Fig. 3:**
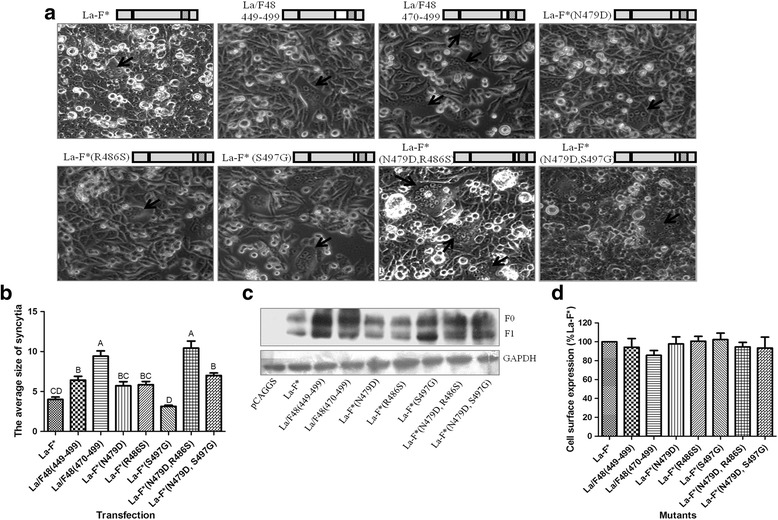
Identification of specific amino acids involved in fusogenic activity. **a** Syncytium formation in BHK-21 cells co-transfected with La-F* and La-HN. **b** Quantitative analysis of the syncytia in (**a**). Statistical significance is designated with lowercase letters (*P* < 0.05) or capital letters (*P* < 0.01). **c** Proteolytic cleavage of the F* proteins in transfected BHK-21 cells was analyzed by Western blot. GAPDH was used as a control. **d** Surface expression of the F* proteins in transfected BHK-21 cells by flow cytometry analysis

Using Western blot analysis, we evaluated the cleavage efficiency of the F* proteins. These F* mutants were partially cleaved into F_1_ subunit in the transfected cells, but there were no significant differences between them (Fig. 3c). These results indicated that the cleavage efficiency of the F* protein was not correlated with fusion. Generally, cleavage of the NDV F protein is necessary for triggering membrane fusion [21]. However, previous studies showed that the recombinant virulent NDV strain with an avirulent Fcs “GRQGR↓L” did not produce syncytia, although the F protein was cleaved, suggesting that other factors might be implicated in membrane fusion in addition to Fcs [14, 22]. We further examined whether the fusion activity induced by the F* mutants was associated with their expression levels on the cell surface. Fluorescence-activated cell sorting (FACS) analysis was performed with an anti-NDV polyclonal antibody in the F* construct-transfected cells, and these cells were compared to those with La-F*. The La-F* mutants showed similar mean fluorescence intensities to wild type F* protein, ranging from 91% to 103% (Fig. 3d). These results suggested that all the mutant F* proteins retained their ability to be transported efficiently to the cell surface, and the loss of fusion activity was not due to decreased cell surface expression level. Several reports have shown that regardless of whether the amino acid mutation in the NDV F protein HR2 domain was conservative or nonconservative, it did not significantly inhibit protein surface expression [23, 24].

Generally, virulent F protein with a virulent Fcs can trigger the fusion activity, and those with an avirulent Fcs fail to induce membrane fusion. In our study, the Fcs “RRQRR↓L” is a deduced avirulent Fcs composed of four basic residues with residue L at position 117 (L^117^) of the F protein. The virulent Fcs usually possesses residue F at position 117 (F^117^), which is required for membrane fusion [25]. Therefore, the F48-F* protein was not expected to induce syncytia. However, we found that the F48-F* protein strongly induced syncytia, whereas the La-F* protein slightly induced syncytia. A previous study showed that residue F mutated to L at position 117 blocked syncytium formation of strain Beaudette C (BC) with the Fcs “RRQKR↓F” in transfected cells [26]. This is inconsistent with our results that either residue F^117^ or L^117^ at the F48-F* enables induction of fusogenic activity. Furthermore, the mutated F48-F with the Fcs “RRQKR↓L” could also induce syncytium formation (data not shown), indicating that the basic residue K or R at position 115 of Fcs has no effect on fusion. These findings may be due to the low amino acid identity (92%) of the F protein between strains F48E9 and BC compared to the strikingly high amino acid identity (98%) between strains LaSota and BC. This suggests that some amino acids in the F proteins of different strains affect the membrane fusion in addition to the Fcs.

The F protein of NDV is a type I membrane protein that forms homotrimers during initial protein folding [2, 27, 28]. Region 118–499 of F contains part of the F_1_ subunit that has sequence elements important in fusion, including HR1 and HR2 [29]. HR1 and HR2 bind each other in an anti-parallel manner as a coiled-coil structure and form the so-called six-helix trimer to drive the merger of viral and target cell membranes [2, 30–32]. The coiled-coil structure is encoded by a seven-residue repeat denoted [abcdefg]_n_, which typically has hydrophobic residues at a and d and polar/charged residues at e and g. The amino acid residues at c, e, and f are located on the outside region of the coiled coil and would not have any effect on the overall structure [33, 34]. Using LearnCoil-VMF analysis [35, 36], we found that N479D and R486S at position f of the coiled-coil structure promoted fusion activity in La-F*, and S497G at position c did not. A previous report showed that the HR1 and HR2 derived from a combination of the F48E9 and avirulent Changchun strains yielded heterotrimer formation, which is required for membrane fusion, despite nine amino acid differences at the positions 145; 139, 461 and 497; 456; 451, 479 and 486; and 192 located at the b, c, e, f, and g sites of the coiled-coil structure [33]. The HR2 of La-F* with the single mutant N479D or R486S or the double mutant (N479D, R486S) may form more stable six-helix bundle trimers with HR1 to promote membrane fusion.

Finally, we examined the role of two amino acids at positions 479 and 486 of F48-F*, wild-type F48-F and La-F in fusogenic activity. These two residues in the F48-F* were mutated to 479 N and 486R of La-F*. The results showed that the mutant F48-F*(D479N, S486R) did not affect membrane fusion compared to F48-F* in transfected BHK-21 cells. Furthermore, the mutants F48-F (D479N, S486R) and La-F (N479D, R486S) did not significantly change the fusogenic activity compared to wild-type F48-F and La-F (data not shown). The results demonstrated that the two mutations affecting fusogenic activity depended on the unique motif “RRQRR↓L”. In addition, although the single mutations La-F*(N479D) and La-F*(R486S) increased membrane fusion activity, their fusion index did not reach that induced by F48-F*. However, the fusion level caused by a substitution construct La-F*/F48(118–499) was similar to that of F48-F* (data not shown), suggesting that except for residues D479 and S486, other amino acids in the 118–499 region of F48-F* might act synergistically to achieve the high level of fusion induced by La-F*. A recent study reported that the mutation N403D in the F protein of mesogenic strain BC with the avirulent Fcs “GRQGR↓L” substantially reduced the fusion activity [17]. Another report showed that the single mutation T458D or G459D in the HR2 region of the F protein in the virulent G7 strain enhanced cell fusion [24]. Taken together, these data suggest that in addition to Fcs, the amino acids of the F protein contribute to fusogenic activity with conditional limitations, including strain, virulence, and cleavage motif sequence. Future studies should examine the effects of the two amino acids at positions 479 and 486 with the unique Fcs motif on virological characteristics by using recombinant virulent and avirulent viruses.

## Abbreviations

aa: Amino acid
FACS: Fluorescence-activated cell sorting
GAPDH: Glyceraldehyde-3-phosphate dehydrogenase
HR1: Heptad repeat 1
HR2: Heptad repeat 2
ICPI: Intracerebral pathogenicity index
NDV: Newcastle disease
